# Effect of Complex Venous Outflow Drainage Reconstruction on Postoperative Graft Function in Right-Lobe Living Donor Liver Transplantation

**DOI:** 10.3390/jcm14062005

**Published:** 2025-03-16

**Authors:** Hakan Kilercik, Sami Akbulut, Ahmed Elsarawy, Sema Aktas, Utku Alkara, Sinasi Sevmis

**Affiliations:** 1 Department of Anesthesiology and Reanimation, Gaziosmanpasa Hospital, Faculty of Medicine, Istanbul Yeni Yuzyil University, 34010 Istanbul, Turkey; hakankilercik@hotmail.com; 2 Department of Surgery and Liver Transplant Institute, Faculty of Medicine, Inonu University, 44280 Istanbul, Turkey; 3 Department of Surgery and Organ Transplantation, Gaziosmanpasa Hospital, Faculty of Medicine, Istanbul Yeni Yuzyil University, 34010 Istanbul, Turkey; amsarawy@gmail.com (A.E.); semaakt@gmail.com (S.A.); ssevmis@yahoo.com (S.S.); 4 Department of Radiology, Gaziosmanpasa Hospital, Faculty of Medicine, Istanbul Yeni Yuzyil University, 34010 Istanbul, Turkey; utkualkara@yahoo.com

**Keywords:** liver transplantation, living donor liver transplantation, complex venous outflow reconstruction, artificial vascular graft, liver graft survival

## Abstract

**Background**: Living donor liver transplantation (LDLT) is the predominant transplantation technique in regions with low rates of deceased donation. Right-lobe grafting is adopted in most clinical and radiological donor/recipient scenarios. Due to the considerable variations in right-lobe hepatic venous anatomy, many techniques have been used over the years for the purpose of appropriate venous outflow reconstruction during the recipient procedure. In this paper, we present the technical details and consequences of a complex venous outflow reconstruction model (CORM) based on experience, and the long-term patency results obtained using the model. **Methods**: Data of patients with end-stage liver disease who underwent LDLT between 21 December 2017 and 29 November 2022 were prospectively collected and retrospectively reviewed. The nomenclature of CORM was assigned when three or more hepatic vein anastomoses were performed. Patients with CORM (CORM group; *n* = 69) were compared with non-CORM patients (non-CORM group; *n* = 130) in terms of demographic, pre- and postoperative clinical, and follow-up features. **Results**: Sixty-nine recipients had three or more separate outflow reconstructions (RHV, RIHV, and one or more anterior sectoral veins); these constituted the CORM group. The estimated graft volume of the CORM group was significantly lower than that of the non-CORM group (833 vs. 898; *p* = 0.022), and the mean GRWR was also significantly lower (1.1 vs. 1.2; *p* = 0.004). CORM cases showed longer anhepatic phases, as well as longer times for cold and warm ischemia, than non-CORM cases (63 vs. 51 min, 46 vs. 38 min, and 48 vs. 33 min, *p* < 0.001), though no difference was found with respect to total operative duration. There were no statistical differences between the two groups with respect to rates of in-hospital re-exploration, length of ICU stay, or length of total hospital stay. Graft survival rates at 1 year, 3 years, and 5 years were 88.1%, 83.3%, and 83.3%, respectively, in the CORM group, and 82.9%, 80.2%, and 70.6%, respectively, in the non-CORM group (*p* = 0.167). **Conclusions**: Performing three or more CORMs in right-lobe LDLT is not associated with inferior outcomes, either with regard to perioperative variables or to patient and graft outcomes. Right-lobe graft with complex venous anatomy from a living donor should not be a determinant factor for donor exclusion.

## 1. Introduction

Since Starzl and colleagues [1] conducted the first successful liver transplantation in 1967 at the University of Colorado, liver transplantation has become the gold-standard treatment for many liver diseases, including end-stage liver disease, acute liver failure, hepatocellular carcinoma, and pediatric inherited metabolic disorders [2]. In developed Western countries, liver grafts obtained from deceased donors are mostly used. In underdeveloped countries, liver grafts are obtained from living donors. In other words, in many parts of the world, including Turkey and other Asian countries, living donor liver transplantation (LDLT) is the only treatment option available to patients with end-stage liver disease, due to an ongoing organ shortage.

In a significant proportion of liver recipients, a right-lobe LDLT graft is procured to meet the proper graft-to-recipient weight ratio (GRWR) and to avoid small-for-size syndrome [3]. Among practitioners at transplant centers, the inclusion or exclusion of the middle hepatic vein within the procured right-lobe graft is a long-lasting controversy [4,5]. However, the establishment of an efficient hepatic venous outflow drainage model is crucial for the proper functionality and durability of the partial transplanted liver graft. Consequently, in cases of right-lobe graft without middle hepatic vein, the reconstruction of significant middle hepatic vein tributaries such as segment V (V5) and segment VIII (V8) veins is an integral step to avoid congestion in the anterior sector. In addition, in some grafts, additional inferior right hepatic veins (IRHVs) may need reconstruction to ensure proper drainage of the posterior sector [6,7].

Although there is no consensus yet, it is believed that the optimal outflow drainage model for right-lobe LDLT should include the IRHVs and anterior sector veins (segments V and VIII) that are ≥ 4–5 mm in diameter [7,8,9,10,11]. When creating a venous outflow drainage reconstruction model, factors such as a high model for end-stage liver disease (MELD) score, a small GRWR ratio, an elderly donor, the volumetric anterior sector size being larger than the posterior sector, and a dominant middle hepatic vein must be taken into account [10]. Many reconstruction techniques have evolved and been named in grafts involving multiple need-to-reconstruct outflow venous channels, including either incorporation into a single intervening venous conduit, or separate anastomoses to the inferior vena cava via venous allografts or synthetic grafts [12]. However, terms such as common large-opening, all-in-one, quilt unification venoplasty, and fence conduit have also been used, based on the technical features of the drainage model [8,13]. In the present case-control study, we investigated the effect of a complex outflow reconstruction model (CORM) on clinical and intraoperative hemodynamic parameters in right-lobe LDLT.

## 2. Materials and Methods

### 2.1. Type, Duration, and Location of Study

After obtaining IRB approval, the prospectively collected electronic medical data of patients (*n* = 281) who underwent LT between 21 December 2017 and 29 November 2022 were retrospectively reviewed. The main goal of this study was to analyze the effect of CORM on fluid resuscitation, blood product transfusion, changes in intraoperative hemodynamic parameters, surgical difficulties, and post-operative complications; for this reason, pediatric LT (*n* = 53), deceased donor LT (*n* = 26), and left-lobe LDLT (*n* = 3) were excluded from the study.

### 2.2. Preoperative Evaluation

Preoperative assessment of the donor liver vasculature was conducted using contrast-enhanced multidetector computed tomography (MDCT) of the upper abdomen. At our center, eligible donors must be ≥18 years old and have a minimum of 30% future liver remnant (FLR) after the proposed right hepatectomy. If preoperative imaging reveals steatosis exceeding 10%, a liver biopsy is performed to confirm that the fatty liver content does not surpass this threshold; otherwise, the living liver donor candidate is deemed ineligible.

Planning for living donor hepatectomy is based on key factors, including donor age, remnant liver volume percentage (RLV), and histopathological hepatosteatosis. In this regard, our center follows the widely accepted algorithm proposed by Prof. Sung-Gyu Lee [10] of the Asan Medical Center, which serves as a standardized guideline for donor selection in LDLT.

### 2.3. Surgical Procedures

All recipients received a right-lobe liver graft without the inclusion of the whole middle hepatic vein. All surgical and anesthetic procedures were carried out by the same team. Donor hepatectomy was performed using the classic hanging maneuver. The demarcation plane was delineated by vascular temporary occlusion and aided by intraoperative ultrasonography in some cases. Parenchymal transection was accomplished using a Cavitron Ultrasonic Surgical Aspirator (CUSA Excel, Valleylab Inc., Boulder, CO, USA) or a shearing device aided by ties and clips, as appropriate. The transection plane was carefully maintained adjacent to the middle hepatic vein, and all significant tributaries were meticulously dissected, isolated, and securely ligated using Hem-o-lok clips to ensure vascular and biliary integrity. 

During the backtable graft preparation, all anterior sectoral (segment V and VIII) or posterior sectoral veins (IRHVs) were reconstructed if one or more of the following factors were met: (i) diameter ≥ 4–5 mm, measured preoperatively and confirmed on the backtable; (ii) significant flow demonstrated during flushing on the backtable according to the surgeon’s experience; (iii) an estimated or actual GRWR < 0.8.

Recipient hepatectomy was performed without total vascular exclusion, and a porto-systemic shunt was not adopted in all patients. Graft implantation was performed under partial lateral-caval clamping. Anterior sectoral veins, if reconstructable, were anastomosed proximally in an end-to-end fashion to a Y-shaped polytetrafluoroethylene (PTFE) single graft, or one-by-one via separate grafts, then distally to the inferior vena cava (at middle hepatic vein–left hepatic vein confluence) in an end-to-end fashion. IRHVs were anastomosed directly to the accessible segment of the inferior vena cava in an end-to-side fashion. Graft RHV was anastomosed to the inferior vena cava in an end-to-side fashion using continuous 5/0 Prolene sutures. Following completion of venous outflow anastomoses, the graft was reperfused after the portal vein anastomosis was secured. Right hepatic artery anastomosis was performed after graft reperfusion in an end-to-end fashion using interrupted 7/0 or 8/0 Prolene sutures. Biliary reconstruction was conducted after arterial anastomosis.

Graft patency and flow were confirmed by intraoperative ultrasonography on day zero, by daily Doppler ultrasonography for the following week, and according to the clinical/laboratory profile thereafter. All patients received triple immunosuppressive therapy (methylprednisolone, tacrolimus, and mycophenolate mofetil), as previously described [14]. Anticoagulant therapy started with low-molecular-weight heparins (LMWHs) following the normalization of the coagulation profile and platelet count. At POD7, low-molecular-weight heparin was discontinued, and acetylsalicylic acid (100 mg/day) was initiated for lifelong maintenance [14].

### 2.4. Intraoperative Hemodynamic Monitoring

During liver transplantation, intraoperative hemodynamic monitoring was performed using PiCCO (Pulse Contour Cardiac Output) and MostCare PRAM (Pressure Recording Analytical Method) systems to ensure accurate assessment of cardiac function, vascular resistance, and fluid responsiveness. All recipients had a central venous catheter (CVC) and invasive arterial blood pressure monitoring put in place prior to the transplantation procedure. The PiCCO system, which utilizes transpulmonary thermodilution and pulse contour analysis, was used to monitor cardiac index (CI), stroke volume variation (SVV), and systemic vascular resistance index (SVRI). Similarly, the MostCare PRAM system provided continuous real-time hemodynamic data without the need for external calibration [15]. Hemodynamic parameters were recorded at predefined time points, including pre-anhepatic, anhepatic, and post-reperfusion phases. Fluid management was guided based on dynamic preload indices, mean arterial pressure (MAP), and central venous pressure (CVP). The choice between crystalloids, colloids, and vasoactive drugs was determined with reference to real-time hemodynamic trends, to maintain optimal organ perfusion and systemic stability throughout the procedure [15].

### 2.5. Definition of Groups and Parameters

In this study, the CORM nomenclature was assigned if three or more hepatic venous outflow reconstruction (i.e., ≥3 venous orifices), including the right hepatic vein, were performed. The study cohort was divided into two groups: a CORM group (≥3 venous reconstructions) and a non-CORM group (<3 venous reconstructions). Both groups were compared in terms of living liver donor features (age, gender, ABO blood groups, body mass index (BMI), hemoglobin (HB), platelet count (PLT), sodium (Na), aspartate aminotransferase (AST), alanine aminotransferase (ALT), prothrombin time (PT), international normalized ratio (INR), albumin, total and direct bilirubin, protein C, protein S, and creatinine). The two groups were also compared with respect to recipient features (age, gender, MELD score, Child score (A, B, C), underlying diseases, hepatitis B virus (HBV), hepatitis C virus (HCV), alcoholism, cryptogenic stroke, autoimmune disease, metabolic dysfunction-associated steatotic liver disease (MASLD), hepatorenal syndrome (HRS), ascites (>1 L encountered upon exploration), hepatocellular carcinoma (HCC), ejection fraction, pulmonary artery pressure (PAP), portal vein thrombosis (PVT), antithrombin III (AT-III), fibrinogen, cold ischemia time (CIT), warm ischemia time (WIT), anhepatic phase, operation time, GRWR, graft volume, fresh frozen plasma (FFP), packed red blood cells (PRBC), intraoperative crystalloids, intraoperative urine output, total bilirubin at postoperative day 30 (POD30), hospital stay, length of stay in intensive care unit (ICU), re-exploration, 30-day mortality, and 1-, 3-, and 5-year survival, as well as a follow-up. Graft loss was defined as either death due to graft dysfunction or a need for transplantation, and graft survival was calculated from the date of transplantation to the date of irreversible graft failure. The follow-up was defined as the time from the date of liver transplantation to the date of the last outpatient clinic control or the date of the patient’s death.

### 2.6. Study Protocol and Ethics Committee Approval

This descriptive and cross-sectional study involving human participants was in accordance with the ethical standards of institutional and national research committees and with the Helsinki Declaration of 1964 and its later amendments or comparable ethical standards. Ethical approval was obtained from the IRB of Inonu University for Non-Interventional Clinical Research (Approval date and no: 2023/5356). To improve the quality of reporting of observational studies, the STROBE (Strengthening the reporting of observational studies in epidemiology) standard was used [16]

### 2.7. Statistical Analysis

Statistical analysis was performed using SPSS version 25.0 (IBM SPSS Statistics, Amarok, NY, USA). Quantitative (continuous, numerical) variables were expressed in the form of Mean (SD) and were compared using Student’s T test or the Mann–Whitney U test as appropriate. Qualitative (categorical) variables were expressed in the form of number (*n*) and percentage (%); these were compared using Fisher’s exact test. Overall survival rates were estimated using Kaplan–Meyer estimates and compared using log-rank testing. A *p* value of <0.05 was considered statistically significant.

## 3. Results

### 3.1. General Assessment of Recipients’ Features

Between 21 December 2017 and 29 November 2022, 199 adult patients (≥18 y) were subjected to right-lobe LDLT at our institution after the exclusion criteria were applied. Median (95% CI) values for age, BMI, and MELD score were 53 (52–56), 26.8 (26–28), and 14 (14–16), respectively. Median (95% CI) values for intraoperative crystalloid use and intraoperative urine output were 5500 mL (5350–6000) and 2200 mL (2000–2400), respectively. The male-to-female ratio was 67/132 (33.7/66.3%). The indications of LDLT were HBV (*n* = 52; 26.1%), MASLD (*n* = 40; 20.1%), cryptogenic stroke (*n* = 32; 16.1%), alcoholism (*n* = 18; 9%), autoimmune disease (*n* = 17; 8.5%), HCV (*n* = 7; 3.5%), and other etiologies (*n* = 33; 16.6%). Most of the patients (*n* = 135; 67.8%) were within the Child B category, with 41 patients (20.6%) being within the Child C category. According to the CORM anastomosis design, 69 patients (34.7%) constituted the CORM group and 130 (65.3%) constituted the non-CORM group. Detailed data of the entire study group are given in Table 1. Table 2 shows the frequency of hepatic venous anatomical variations within the CORM group and the consequent design of outflow reconstruction. The most frequent variation (*n* = 35/69, 51%) was the presence of two significant middle hepatic vein (anterior sectoral) tributaries, followed by one significant anterior sectoral vein and one IRHV (*n* = 17/69; 25%). A Y-shaped PTFE graft was employed in 37 patients.

### 3.2. Comparison of CORM and Non-CORM Groups

Table 3 shows a comparison of the CORM and non-CORM groups according to demographic and clinical characteristics of living liver donors. Living liver donors in the CORM group showed lower median BMI (23.7 vs. 25; *p* = 0.011) and HB (14.5 vs. 15.1; *p* = 0.013) values, and these differences were statistically significant. No statistically significant differences between groups were recorded with respect to other donor characteristics such as gender (*p* = 0.144), ABO group matching (*p* = 0.277), and the abovementioned biochemical blood parameters. In other words, features of living liver donors did not influence group formation, resulting in homogeneous groups in this regard.

Table 4 shows a comparison of the CORM and non-CORM groups according to demographic and preoperative clinical characteristics of recipients. The incidence of HCC was higher among the non-CORM group (26% vs. 13%), and this difference demonstrated borderline statistical significance (*p* = 0.050). No statistically significant differences were recorded between groups with respect to recipients’ demographic characteristics (age, gender, BMI), clinical characteristics (Child score, MELD score, underlying diseases, HCC, ejection fraction, PAP, ascites, HRS, and PVT), or the abovementioned biochemical blood parameters.

Table 5 shows a comparison of the CORM and non-CORM groups in terms of intraoperative clinical characteristics of recipients. The median anhepatic phase (63 vs. 51; *p* < 0.001), CIT (46 vs. 38; *p* < 0.001), and WIT (48 vs. 33; *p* < 0.001) times were prolonged in the CORM group, compared with the non-CORM group. On the other hand, intraoperative use of FFPs (1 vs. 2; *p* = 0.011), procured graft volume (833 vs. 898; *p* = 0.022), and GRWR (1.1 vs. 1.2; *p* = 0.004) were higher in the non-CORM group. No statistically significant differences were found between groups in terms of intraoperative PRBC use (*p* = 0.330), intraoperative crystalloid use (*p* = 0.659), intraoperative urine output (*p* = 0.559), or operation time (*p* = 0.343).

Table 6 shows a comparison of the CORM and non-CORM groups in terms of postoperative characteristics of recipients. No statistically significant differences were found between the groups with respect to POD30 total bilirubin level (*p* = 0.483), hospital stay (*p* = 0.426), ICU stay (*p* = 0.304), re-exploration (*p* = 0.569), 30-day mortality (*p* = 1.000), 365-day mortality (*p* = 0.586), or follow-up (*p* = 0.074).

### 3.3. Comparison of CORM and Non-CORM Groups Based on Survival

**Figure 1:**
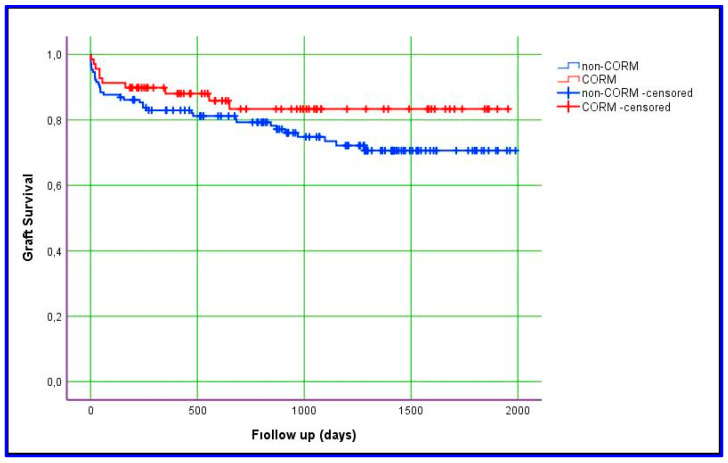
Kaplan–Meier estimation of graft survival.

**Figure 2:**
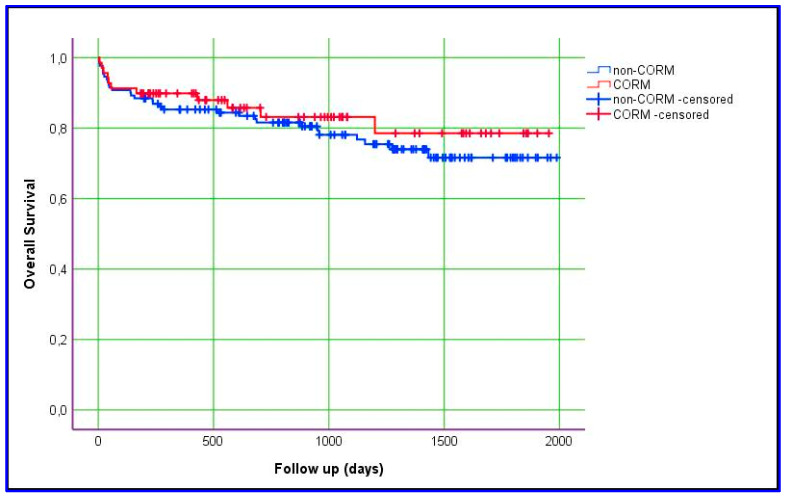
Kaplan–Meier estimation of overall patient survival.

Kaplan–Meier estimation was used to evaluate differences between the groups with respect to graft survival and overall survival. In this study, we obtained data for overall and graft survival rates at 1, 3, and 5 years. The mean ± SD value for overall survival in the CORM group was 1635 ± 87 days, compared with 1575 ± 66 days in the non-CORM group. The overall survival rates were 89.9%, 83.1%, and 78.5% at 1, 3, and 5 years, respectively, in the CORM group; in the non-CORM group, they were 85.3%, 80.5%, and 71.6%, respectively. No statistically significant difference was observed between the groups (*p* = 0.461). Similarly, the mean ± SD value for graft survival in the CORM group was 1668 ± 83 days, compared with 1528 ± 69 days in the non-CORM group. The graft survival rates were 88.1%, 83.3%, and 83.3% at 1, 3, and 5 years, respectively, in the CORM group; in the non-CORM group, they were 82.9%, 80.2%, and 70.6%, respectively. No statistically significant difference was observed (*p* = 0.167). Although the differences were not statistically significant, the CORM group exhibited relatively better survival outcomes, likely due to the effectiveness of venous outflow drainage (Figure 1 and Figure 2) (Table 7).

### 3.4. Comparison of CORM and Non-CORM Groups Based on Postoperative Early Complications

Table 6 indicates that no significant differences were observed between the groups in the early postoperative period (first month) with respect to hospital stays, bilirubin levels, or reoperation rates. Graft loss resulted from primary non-function in four cases and from hepatic artery thrombosis in two cases within the non-CORM group, whereas only primary non-function was noted in two cases in the CORM group. Moreover, there were no notable differences between the groups regarding postoperative ascites drainage or temporal variations in liver function tests.

## 4. Discussion

Liver transplantation has become established as the only curative pathway for end-stage liver disease and certain hepatic malignancies, as well as for various metabolic disorders. Due to the worldwide organ shortage situation, LDLT has become the primary therapeutic option in many regions [17]. In adult-to-adult LDLT, right lobe procurement is the standard of care in most clinical encounters, so as to achieve a secure GRWR and to avoid small-for-size syndrome [18,19].

Considering the principle of double equipoise, which prioritizes the safety of both donor and recipient, the dilemma of including or excluding the middle hepatic vein has emerged and been thoroughly discussed in the transplant scientific community in recent years. It is now widely agreed that securing an efficient graft outflow via meticulous reconstruction of all right-lobe venous territories is crucial for the recipient’s best outcome [20,21].

In regions where LDLT without middle hepatic vein is predominantly practiced, the finding of a right-lobe liver graft with a complex venous outflow anatomical variation is frequently encountered [8]. Exclusion of a donor because of complex vascular anatomy may deprive many end-stage liver disease patients of a chance of curative LT. This venous outflow complexity is the result of the numerous variations in anterior draining to the middle hepatic vein and in posterior draining to the inferior vena cava sectoral veins, as well as the main right hepatic vein [6,22]. From a surgical-anatomical standpoint, numerous variations in the medical hepatic vein and its territories have been described in previous reports, with double V5, double V8, duplicated middle hepatic vein and other different combinations being reported [23,24].

In this setting of multiple venous outflow orifices such as IRHVs, V5, and V8, some transplant centers have adopted the all-in-one technique by relaying all sectoral branches via one intervening allograft [8] (sometimes referred to as a neo-middle hepatic vein) [22] or a synthetic conduit to the inferior vena cava. At our transplant center, we have systematically performed one-by-one venous anastomoses, but in situations where we have encountered two reconstructable fairly close venous orifices, a Y-shape Dacron or PTFE graft has been employed.

In the present study, the CORM group showed venous outflow patterns ranging from double anterior sectoral veins to as many as four or five hepatic veins from anterior and posterior sectors, in addition to the right hepatic vein. The most frequent venous outflow anatomical entity among the CORM group was double anterior sectoral veins (35 cases, 51%), along with RHV (i.e., three cases of graft venous anastomotic orifices). A Y-shaped synthetic graft was a suitable conduit in most (30/35) of these cases. Our findings, as well as our surgical approach, are consistent with the study of 141 LDLT recipients conducted by Guo and colleagues [25] in which 55 patients (55% of the reconstructed middle hepatic vein group) were found to harbor double anterior sectoral veins; for 44 of them, a Y-shaped graft was a suitable reconstruction design [25].

The types and availability of vascular graft materials used are crucial components of a complex venous outflow reconstruction model. In cases of such complex venous anatomy, outflow reconstruction is a domain characterized by considerable technical innovation and expertise [12]. In areas where there is an insufficient level of organ and tissue donation by deceased donors, there is also a shortage of autologous vascular grafts. Consequently, the use of synthetic grafts such as polyethylene terephthalate (Dacron) and PTFE are the principal techniques used for venous outflow reconstruction of partial liver grafts [26,27]. In areas with average rates of deceased donation, venous conduits are procured and adopted. Due to inadequate access to vascular grafts obtained from deceased donors, we routinely use synthetic vascular grafts for venous reconstruction. In recent years, we have preferred to use Dacron grafts, due to their flexible structure, manipulability, and easy accessibility.

Despite these benefits, it has been argued, based on previous reports, that the use of Dacron grafts might be associated with a higher risk of infection due to their large pores, a situation that could raise the risk of liver graft infection; in addition, there is a higher possibility of bile leakage (biloma) into the surgical site, especially in the setting of LDLT, as well as an immunosuppressed state. This has led some centers to reconsider using Dacron vascular grafts [26,28,29]. Koc and colleagues [30] have shown that bile leakage is an independent risk factor for vascular graft infection, but one of the most important findings of this study is that PTFE grafts are three times more likely to be infected than Dacron grafts. Contrary to the available evidence [28], the results of this study are nonetheless consistent with our clinical experience, as we have not experienced increased incidence of infectious complications secondary to Dacron vascular grafts (unpublished data).

According to our experience, the CORM model secures the drainage of all significant sectoral veins with a prolongation of intraoperative parameters which is statistically—but not clinically—significant, including AHF (*p* < 0.001), CIT (*p* < 0.001), and WIT (*p* < 0.001). Moreover, the results obtained during our study period with respect to operative duration, intraoperative PRBC transfusion, ICU stays, and hospital stays are all well aligned with the quality benchmarks of LDLT published recently [31]. We think that, because the grafts of the patients in the CORM group were drained optimally, the results were at least as good as those of the patients in the non-CORM group. Statistically, no differences were evident between our study groups in terms of 1-year graft or 1-year overall survival parameters. In fact, long-term graft survival and overall survival rates appear to be relatively better in the CORM group (Figure 1 and Figure 2). Our finding that a prolongation of the CIT in favor of meticulous outflow reconstruction did not negatively impact the graft and survival parameters augments the conclusion by Goja and colleagues [32], who noticed that CIT protraction in a group of LDLT recipients in whom middle hepatic vein reconstruction was made by synthetic vascular conduits did not negatively impact long-term graft or survival outcomes.

Taking into consideration the fact that graft regeneration and subsequent enlargement might alter the orientation of outflow reconstruction and hence impede the outflow [20], we pursued the principle of one-by-one rather than all-in-one. However, data from many centers support the use of the all-in-one technique, and there has been as yet no randomized controlled trial to bring this debate to an end. Taha A and colleagues carried out a comparison among a cohort of 40 recipients, all of whom had anterior sectoral reconstruction using PTFE, either one-by-one or in the form of a common conduit; they found no statistically significant differences in terms of graft early recovery or outcome [22].

Serum total bilirubin level after one month of LDLT has been employed as a surrogate early indicator of graft stability and outcome [33]. In our study, total bilirubin level at the POD30 among the CORM group showed no difference from the other group, indicating an absence of negative graft outcome when multi-outflow reconstructions were separately performed. Moreover, no difference was evident in terms of 1-year graft or 1-year overall survival parameters. Interestingly, the median graft weight (*p* = 0.002) and median GRWR (*p* = 0.004) were both lower among the CORM group, with statistical significance. Our explanation is that there might be a tendency for the transplant surgeon to reconstruct all venous outflow orifices in cases of relatively small grafts, to obviate possible small-for-size syndrome.

### Limitations

The present study has some limitations. Its retrospective nature raises the possibility of inherent bias. In addition, an absence of intraoperative values for graft inflow and outflow dynamics, a shortage of data about vascular graft patency rates, and a small sample size should also all be noted. Future studies might focus on a randomized control trial across multiple transplant centers, to determine whether procurement of a non-middle hepatic vein right lobe is needed, thereby addressing many questions and concerns.

## 5. Conclusions

A right-lobe graft with complex venous outflow anatomy is not associated with an inferior graft outcome if surgery and outflow reconstruction are carried out with precision. Reconstruction of a complex outflow venous anatomy is feasible without significant clinical implications during an LT procedure.

## Figures and Tables

**Table 1:** Recipient characteristics of the entire study group.

Features	Result
Age (years)	53 (52–56)
Gender (male/female (%))	132/67 (66.3/33.7)
Child score (A/B/C (%))	23/135/41 (11.6/67.8/20.6)
HCC (*n* (%))	43 (21.6)
BMI (kg/m^2^)	26.8 (26–28)
MELD score	14 (14–16)
Ejection fraction (%)	65 (65–70)
PAP (mmHg)	30 (30–35)
HB	10.6 (10–11.3)
PLT	81 (73–91)
Creatinine	0.78 (0.74–0.83)
Na	137 (137–138)
Albumin	3.2 (3.1–3.3)
AST	52 (49–59)
ALT	35 (32–40)
Total bilirubin	1.8 (1.6–2.1)
Direct bilirubin	0.9 (0.9–1.2)
PT	16.1 (15.7–16.7)
INR	1.4 (1.3–1.4)
Fibrinogen	124 (124–132)
AT-III	54 (50–57)
Protein S	66 (65–70)
Protein C	45 (42–49)
Intraop PRBCs	2 (2–3)
Intraop FFPs	2 (2–3)
Intraop crystalloids (mL)	5500 (5350–6000)
Intraop urine output (mL)	2200 (2000–2400)
Anhepatic phase (min)	56 (53–60)
CIT (min)	41 (40–44)
WIT (min)	40 (39–42)
Graft volume (gr)	876 (846–905)
GRWR	1.2 (1.2–1.4)
Operation time (hour)	6 (6–7)
PVT (*n* (%))	33 (16.6)
HRS (*n* (%))	37 (18.6)
Ascites (*n* (%))	175 (87.9)
Total bilirubin (POD30)	0.7 (0.6–0.8)
Hospital stay (days)	14 (13–15)
ICU stay (days)	1 (1–2)
Re-exploration (*n* (%))	32 (16.1)
30-day mortality (*n* (%))	10 (5.0)
365-day mortality (*n* (%))	25 (12.6)
Follow-up (days)	920 (860–1024)

Quantitative variables are given as median values (95% CI), and qualitative variables are given as numbers (%). BMI—body mass index; MELD—Model for end-stage liver disease; HCC—hepatocellular carcinoma; HRS—hepatorenal syndrome; PVT—portal vein thrombosis; AT-III—antithrombin III; INR—international normalized ratio; PT—prothrombin time; AST—aspartate aminotransferase; ALT—alanine aminotransferase; HB—hemoglobin; PLT—platelet count; Na—sodium; GRWR—graft-to-recipient weight ratio; CIT—cold ischemia time; WIT—warm ischemia time; FFP—fresh frozen plasma; PRBC—packed red blood cells.

**Table 2:** Details of anastomosis in CORM group (*n* = 69).

Reconstruction Features	Anastomosis Design	Number of Patients
RHV+ V5 and V8, RHV+ V5 (*n* = 2) and RHV + V8 (*n* = 2)	RHV to RHV, V5 and V8 via Y-shape PTFE graft to IVC	30
	RHV to RHV, V5 and V8 via separate PTFE grafts to IVC	5
RHV+ (V5 or V8) + RIHV	RHV to RHV, IRHV to IVC, V5 via PTFE graft to IVC	17
RHV+ RIHVs (*n* = 2)	RHV to RHV, each IRHV separate distal anastomosis to IVC	2
RHV+ V5 + RIHVs (*n* = 2)	RHV to RHV, each IRHV separately to IVC, V5 via PTFE graft to IVC	5
RHV+ V5+ V8 + RIHV	RHV to RHV, IRHV to IVC, V5 and V8 via separate PTFE grafts to IVC	4
	RHV to RHV, IRHV to IVC, V5 and V8 via Y-shape PTFE graft to IVC	4
RHV+ V5+ V8 + RIHVs (*n* = 2)	RHV to RHV, each IRHV separately to IVC, V5 and V8 via Y-shape PTFE graft to IVC	1
RHV+ V5 (*n* = 2) + V8 + RIHV	RHV to RHV, IRHV to IVC, 2 V5 via Y-shape PTFE graft to IVC and V8 via PTFE to IVC	1

RHV—right hepatic vein; V5—segment V vein; V8—segment VIII vein; IVC—inferior vena cava; RIHV—right inferior hepatic vein; MHV—middle hepatic vein; PTFE—polytetrafluoroethylene.

**Table 3:** Comparison of CORM and non-CORM groups in terms of living liver donors’ features.

Features	CORM (*n* = 69)	Non-CORM (*n* = 130)	*p*
Gender (%)			0.144
Male	41 (59.4)	92 (70.8)	
Female	28 (40.6)	38 (29.2)	
ABO matching (%)			0.277
Identical	48 (69.6)	101 (77.7)	
Compatible	21 (30.4)	29 (22.3)	
Age	30 (28–34)	33 (32–35)	0.279
BMI	23.7 (22.6–25)	25 (24.6–25.8)	0.011
HB	14.5 (13.9–15.0)	15.1 (14.9–15.4)	0.013
PLT	252 (238–266)	252 (244–275)	0.992
Creatinine	0.8 (0.8–0.9)	0.8 (0.8–0.9)	0.123
Na	140 (140–141)	140 (140–141)	0.982
Albumin	4.8 (4.8–5.0)	4.7 (4.7–4.8)	0.168
AST	18 (17–20)	19 (19–20)	0.071
ALT	16 (15–20)	18 (17–21)	0.092
Total bilirubin	0.4 (0.4–0.5)	0.5 (0.5–0.6)	0.893
Direct bilirubin	0.2 (0.2–0.3)	0.2 (0.2–0.3)	0.585
Cholesterol (total)	158 (153–167)	164 (157–170)	0.348
Triglycerides	81 (76–90)	96 (85–106)	0.179
PT	12 (11.8–12.2)	11.7 (11.6–12)	0.083
INR	1.0 (1.0–1.1)	1.0 (1.0–1.1)	0.052
Protein C	100 (97–108)	102 (98–108)	0.511
Protein S	88 (84–91)	89 (86–92)	0.412

Quantitative variables are given as median values (95% CI), and qualitative variables are given as numbers (%). BMI—body mass index; INR—international normalized ratio; PT—prothrombin time; AST—aspartate aminotransferase; ALT—alanine aminotransferase; HB—hemoglobin; PLT—platelet count; Na—sodium.

**Table 4:** Comparison of CORM and non-CORM groups in terms of demographic and preoperative features of recipients.

Features	CORM (*n* = 69)	Non-CORM (*n* = 130)	*p*
Age	51 (49–56)	54 (53–57)	0.510
Gender			0.235
Male	42 (60.9)	90 (69.2)	
Female	27 (39.1)	40 (30.8)	
Child score			0.670
A	9 (13)	14 (11)	
B	44 (64)	91 (70)	
C	16 (23)	25 (19)	
Underlying diseases			0.336
HBV	13 (18.8)	39 (30)	
HCV	1 (1.4)	6 (4.6)	
Alcohol	7 (10.1)	11 (8.5)	
MASLD	19 (27.5)	21 (16.2)	
Cryptogenic	12 (17.4)	20 (15.4)	
Autoimmune	5 (7.2)	12 (9.2)	
Others	12 (17.4)	21 (16.2)	
HCC	9 (13)	34 (26)	0.050
BMI	28 (26- 29)	26 (25–27)	0.089
MELD score	16 (14–18)	14 (14–17)	0.336
Ejection fraction (%)	65 (65–70)	65 (65–70)	0.636
PAP (mmHg)	30 (30–35)	30 (30–35)	0.266
HB	11 (10–12)	10.6 (10–11.5)	0.688
PLT	83 (72–101)	79 (68–91)	0.688
Creatinine	0.71 (0.63–0.81)	0.78 (0.75–0.85)	0.127
Na	137 (137–139)	137 (137–138)	0.402
Albumin	3.2 (2.9–3.4)	3.2 (3.1–3.4)	0.905
AST	52 (42–63)	51 (46–59)	0.780
ALT	33 (29–46)	36 (31–40)	0.863
Total bilirubin	1.9 (1.4–2.5)	1.7 (1.5–2.2)	0.736
Direct bilirubin	0.9 (0.7–1.3)	0.9 (0.8–1.3)	0.453
PT	16.2 (15.5–17.7)	16.1 (15.5–16.7)	0.350
INR	1.4 (1.3–1.5)	1.4 (1.3–1.4)	0.292
Fibrinogen	124 (124–134)	124 (124–132)	0.168
AT-III	48 (44–57)	54.5 (50–59)	0.176
Protein S	66 (63–72)	66.5 (65–72)	0.970
Protein C	44 (39–52)	45 (41–49)	0.941
PVT	10 (11.4)	23 (21.6)	0.706
HRS	11 (15.9)	25 (19.2)	0.704
Ascites	61 (88)	114 (88)	1.000

Quantitative variables are given as median values (95% CI), and qualitative variables are given as numbers (%). BMI—body mass index; HBV—hepatitis B virus; HCV—hepatitis C virus; MELD—model for end-stage liver disease; HCC—hepatocellular carcinoma; HRS—hepatorenal syndrome; PVT—portal vein thrombosis; AT-III—antithrombin III; INR—international normalized ratio; PT—prothrombin time; AST—aspartate aminotransferase; ALT—alanine aminotransferase; HB—hemoglobin; PLT—platelet count; Na—sodium; MASLD—metabolic dysfunction-associated steatotic liver disease.

**Table 5:** Comparison of CORM and non-CORM groups in terms of intraoperative variables.

Features	CORM (*n* = 69)	Non-CORM (*n* = 130)	*p*
Intraop PRBCs	2 (2–3)	2 (2–3)	0.330
Intraop FFPs	1 (1–2)	2 (2–3)	0.011
Intraop crystalloids (mL)	5500 (5500–6000)	5650 (5000–6000)	0.659
Intraop urine output (mL)	2250 (1800–2500)	2000 (2000–2300)	0.559
Anhepatic phase (min)	63 (59–70)	51 (47–57)	<0.001
CIT (min)	46 (42–50)	38 (35–42)	<0.001
WIT (min)	48 (43–50)	33 (30–37)	<0.001
Graft volume (gr)	833 (800–887)	898 (855–935)	0.022
GRWR	1.1 (1.1–1.2)	1.2 (1.2–1.4)	0.004
Operation time (hours)	6 (6–7)	6 (6–7)	0.343

Quantitative variables are given as median values (95% CI). GRWR—graft-to-recipient weight ratio; CIT—cold ischemia time; WIT—warm ischemia time; FFP—fresh frozen plasma; PRBC—packed red blood cells.

**Table 6:** Comparison of CORM and non-CORM groups in terms of postoperative features.

Features	CORM (*n* = 69)	Non-CORM (*n* = 130)	*p*
Total bilirubin (POD30)	0.66 (0.45–1)	0.74 (0.42–1.3)	0.483
Hospital stay	15 (15–18)	14 (13–17)	0.426
Re-exploration	13 (19)	19 (15)	0.569
ICU stay (day)	1 (1–2)	1 (1–2)	0.304
30-day mortality	3 (4)	7 (5)	1.000
365-day mortality	7 (10.1)	18 (14.0)	0.586
Follow-up (days)	644 (520–982)	959 (871–1256)	0.074
Graft loss			0.716
Primary non-function	2	4	
Hepatic artery thrombosis	0	2	

Quantitative variables are given as median values (95% CI), and qualitative variables are given as numbers (%). POD—postoperative days; ICU—intensive care unit.

**Table 7:** Comparison of CORM and non-CORM groups in terms of survival rates.

Features	CORM (*n* = 69)	Non-CORM (*n* = 130)	*p*
Overall survival			0.461
1 year	89.9%	85.3%	
3 years	831%	80.5%	
5 years	78.5%	71.6%	
Graft survival			0.167
1 year	88.1%	82.9%	
3 years	83.3%	80.2%	
5 years	83.3%	70.6%	

## Data Availability

The data presented in this study are available upon request from the corresponding author. The ethics committee requests a confidentiality letter for the management of this study’s data due to the inclusion of personal and clinical patient data.
